# Identification of positively selected genes in *Mycobacterium tuberculosis* from southern Xinjiang Uygur autonomous region of China

**DOI:** 10.3389/fmicb.2024.1290227

**Published:** 2024-04-15

**Authors:** Lele Deng, Quan Wang, Haican Liu, Yi Jiang, Miao Xu, Yu Xiang, Ting Yang, Shuliu Yang, Di Yan, Machao Li, Lili Zhao, Xiuqin Zhao, Kanglin Wan, Guangxue He, Xiaokaiti Mijiti, Guilian Li

**Affiliations:** ^1^National Institute for Viral Disease Control and Prevention, Chinese Center for Disease Control and Prevention, Beijing, China; ^2^National Key Laboratory of Intelligent Tracking and Forecasting for Infectious Diseases, National Institute for Communicable Disease Control and Prevention, Chinese Center for Disease Control and Prevention, Beijing, China; ^3^Eighth Affiliated Hospital of Xinjiang Medical University, Urumqi, China; ^4^School of Public Health, University of South China, Hengyang, China

**Keywords:** tuberculosis, *Mycobacterium tuberculosis*, whole genome sequencing (WGS), positive selection, China

## Abstract

**Background:**

Tuberculosis (TB), mainly caused by *Mycobacterium tuberculosis* (*Mtb*), remains a serious public health problem. Increasing evidence supports that selective evolution is an important force affecting genomic determinants of *Mtb* phenotypes. It is necessary to further understand the *Mtb* selective evolution and identify the positively selected genes that probably drive the phenotype of *Mtb*.

**Methods:**

This study mainly focused on the positive selection of 807 *Mtb* strains from Southern Xinjiang of China using whole genome sequencing (WGS). PAML software was used for identifying the genes and sites under positive selection in 807 *Mtb* strains.

**Results:**

Lineage 2 (62.70%) strains were the dominant strains in this area, followed by lineage 3 (19.45%) and lineage 4 (17.84%) strains. There were 239 codons in 47 genes under positive selection, and the genes were majorly associated with the functions of transcription, defense mechanisms, and cell wall/membrane/envelope biogenesis. There were 28 codons (43 mutations) in eight genes (*gyrA*, *rpoB*, *rpoC*, *katG*, *pncA*, *embB*, *gid*, and *cut1*) under positive selection in multi-drug resistance (MDR) strains but not in drug-susceptible (DS) strains, in which 27 mutations were drug-resistant loci, 9 mutations were non-drug-resistant loci but were in drug-resistant genes, 2 mutations were compensatory mutations, and 5 mutations were in unknown drug-resistant gene of *cut1*. There was a codon in *Rv0336* under positive selection in L3 strains but not in L2 and L4 strains. The epitopes of T and B cells were both hyper-conserved, particularly in the T-cell epitopes.

**Conclusion:**

This study revealed the ongoing selective evolution of *Mtb*. We found some special genes and sites under positive selection which may contribute to the advantage of MDR and L3 strains. It is necessary to further study these mutations to understand their impact on phenotypes for providing more useful information to develop new TB interventions.

## 1 Introduction

Tuberculosis (TB), caused by the *Mycobacterium tuberculosis complex* (MTBC), remains a serious public health problem (World Health Organization, 2023). The human TB is mainly caused by *Mycobacterium tuberculosis* (*Mtb*) *sensu stricto* (lineages 1, 2, 3, 4, and 7), *Mycobacterium africanum* (lineages 5 and 6), and newly recognized *Mtb* lineages (lineages 8 and 9) (Gagneux, 2018; Ngabonziza et al., 2020; Coscolla et al., 2021; Freschi et al., 2021). The human-adapted MTBC exhibits obvious phylogeographical population structure (Gagneux, 2018), with lineage 2 (L2) and lineage 4 (L4) strains distributing and spreading globally and others showing a geographical distribution (Brites and Gagneux, 2015; Liu et al., 2021). This suggests the hypothesis that the strains have adapted to local human populations through selective evolution (Gagneux, 2012; Brites and Gagneux, 2015; Liu et al., 2021). Although some social factors influence the epidemiology of *Mtb*, the evidence supporting selective evolution has been reported in many studies (Ameke et al., 2016; Liu et al., 2021). Additionally, other phenotypic features exhibit the selective evolution of *Mtb* strains, for example, transmissibility, virulence, host response, and drug resistance vary in different lineages (Reiling et al., 2013; Portevin et al., 2014). The single mutations driving phenotypic differences have been identified (Chiner-Oms et al., 2022). Selective evolution is an important force in fixing the mutations in bacterial populations, which affect the genomic determinants of these phenotypes (Chiner-Oms et al., 2022).

Positive selection shaping the selective evolution of *Mtb* has been revealed by some studies (Osório et al., 2013; Stucki et al., 2016; Liu et al., 2021). Positive selection indicates that the genes and sites are undergoing selective evolution when the ratio of non-synonymous to synonymous substitutions is greater than 1 (Liu et al., 2021). Several studies showed that, under the positive selection, some genes, especially drug resistance-associated genes (*katG*, *rpoB*, *rpoC*, and *embB*) and genes related to important functions (*lppA*, *esxN*, and *sseA*), resulted in the selective evolution of *Mtb* (Farhat et al., 2013; Zhang et al., 2013; Liu et al., 2021). At the same time, previous studies have reported ongoing positive selection of specific genomic regions, especially in epitopes and essential genes (Comas et al., 2010; Gagneux, 2018). Studies reported that human T-cell epitopes are highly conserved in *Mtb*, suggesting that *Mtb* has adopted a strategy of immune subversion (Comas et al., 2010; Phelan et al., 2016; Gagneux, 2018). While based on the T-cells, the cellular immune system majorly protects the host against *Mtb* during the most stages of the infection, there have been supporting data for the roles of B cells and antibodies in the defense against *Mtb* infection recently (Achkar et al., 2015).

The notifiable TB morbidity was 45.37/100,000 in 2021 in China, ranking second among all statutory infectious diseases (National Health Commission of the People’s Republic of China, 2022). Xinjiang Uygur Autonomous Region is one of the areas with a high TB burden, with a morbidity rate of 87.85/100,000 in 2021 (National Health Commission of the People’s Republic of China, 2022). Several studies explored that the characteristics of *Mtb* in Xinjiang with whole genome sequencing (WGS) have been reported (Anwaierjiang et al., 2021; He et al., 2022; Xu et al., 2022), including *Mtb* population structure (Xu et al., 2022), drug resistance (Anwaierjiang et al., 2021), and virulence (He et al., 2022). To date, the report on detailed analysis of the selective evolution of *Mtb* strains has not been found in this area. Therefore, we collected samples from Southern Xinjiang to explore the positive selection of *Mtb* and to provide some useful information for strengthening TB control.

## 2 Materials and methods

### 2.1 Sample resource

The samples were collected from the TB patients in four TB designated hospitals, including the Eighth Affiliated Hospital of Xinjiang Medical University, Kuqa County Infectious Disease Hospital, Kashgar Pulmonary Hospital, and Wushi County People’s Hospital. The experimental protocol was established (number XJMU8HEC-20161215). Written informed consent was obtained from the study participants. The inclusion criteria for the research subjects are as follows: (i) All bacteriological-confirmed TB cases should be consistent with the TB diagnosis criteria (National Health Commission of the People’s Republic of China, 2017); (ii) The subjects should be residing in local areas; (iii) All subjects must provide qualified samples and ensure that one strain was isolated from one patient. A total of 863 isolates were collected in four hospitals during 2019–2021.

### 2.2 Whole genome sequencing

All strains were sub-cultured on the Löwenstein–Jensen medium from stored samples that were kept in −80°C freezer. The genomic deoxyribonucleic acid (DNA) was extracted using the cetyl trimethyl ammonium bromide (CTAB) method (Li et al., 2022). Then, the libraries of DNA were constructed. The WGS was performed with MGISeq-2000 (read length: 150 bp, sequencing depth: 500×, paired-end sequencing, Supplementary Table S1). The exclusion criteria of strains are as follows: (i) infection with more than one lineage strain; (ii) coverage of less than 95% of the genome with a depth of at least 10×; (iii) Non-*Mtb* strains by blasting the sequence of 16 s RNA.

### 2.3 Sequencing data analysis and lineage identification

TBProfiler software (v4.1.2) was used for single nucleotide polymorphism (SNP) calling and lineage identification based on the 90 barcode SNPs (Phelan et al., 2019; Napier et al., 2020). First, trimmomatic software (v0.39) was used for trimming raw sequences (Bolger et al., 2014). Second, reads were aligned to the H37Rv (NC_000962.3) reference genome using BWA mem (0.7.17, default parameters) (Li, 2013). Third, samtools was used for sorting reads and removing duplicate reads (v1.12, default parameters) (Danecek et al., 2021). Genetic variants were called by bcftools (v1.15) (Danecek et al., 2021) and freebayes (v1.3.6, min mapping quality >30, min coverage >5, min reads >2, GT = 1/1, DP >10, Supplementary Table S1) (Garrison and Marth, 2012). All mentioned steps were conducted by TBProfiler, and vcf files were acquired. The vcf files were annotated using SnpEff (5.1d) (Cingolani et al., 2012).

The vcf files were filtered using bcftools (v1.15) (Danecek et al., 2021). First, indels and insertions were removed, and only SNPs were kept for the next analysis. Second, SNPs in the repetitive regions of the genome were excluded using bcftools, including non-essential *PE/PPE/PE-PGRS* family genes (Phelan et al., 2016), insertion, phage sequences, and mobile genetic elements. Several *PE/PPE/PE-PGRS* family genes were included in this study based on the findings of other study, including *PE-PGRS3*, *PE-PGRS4*, *PE-PGRS17*, *PPE57*, *PPE59*, and *PPE60* (Phelan et al., 2016). We applied specific filtering criteria to ensure the retention of high-quality alignment results, including minimum mapping quality, read coverage, and alignment uniqueness thresholds. All filtered vcf files were merged into a multi-vcf file using bcftools. A multi-fasta file containing all isolates was generated from the multi-vcf file using vcf2phylip (v1.5) (Ortiz, 2019). Gffread software (v0.12.7) was used to extract the coding sequence (CDS) (Pertea and Pertea, 2020).

### 2.4 Construction of phylogenetic trees and principal component analysis

Maximum likelihood phylogenetic trees were constructed using IQtree (v2.0.3, 1,000 bootstraps) (Nguyen et al., 2015). The tree file was visualized in ChiPlot.^1^ The principal component analysis (PCA) of the SNPs was performed using Plink software (v1.90) (Chang et al., 2015).

### 2.5 Selective pressure analysis

The ratios of non-synonymous to synonymous substitution rates (*d_N_/d_S_*) for each gene across all sites were calculated to explore the characteristics of positive selection using PAML software (v 4.9j), with *d_N_/d_S_* being <1, = 1, and > 1, indicating negative selection, neutral evolution, and positive selection, respectively (Yang, 2007). Codeml, one of the programs of PAML, was used for model selection using the maximum likelihood approach. The likelihood ratio tests (LRTs) of each model were calculated by the codeml program and were compared between the null model (M1a, M7: not allow *d_N_/d_S_* > 1) and the alternative model (M2a, M8: allow *d_N_/d_S_* > 1). The statistical significance of LRTs was tested by the chi-squared test (degree of freedom: 2), and the *p*-value indicated the likelihood of the alternative model. The Bayes Empirical Bayes (BEB) was used to identify the sites under positive selection if the LRTs were statistically significant.

The input files included the aligned sequences and the tree files, which are evaluated by Prank (v.170427) (Löytynoja, 2021) and IQtree (v2.0.3), respectively (Nguyen et al., 2015). We extracted CDS sequences for each sample by Gffread software (v0.12.7). Moreover, the sequences of each gene were combined and aligned based on different datasets, including 147 MDR strains, 415 DS strains, 157 L3 strains, 506 L2 strains, and 144 L4 strains. The tree files were constructed based on the alignment sequences of each gene in different datasets.

The Clusters of Orthologous Groups of Proteins (COGs^2^) database was used for classifying the genes and proteins based on their function (Tatusov et al., 2000). SIFT online website^3^ was used to predict the possible impact of an amino acid substitution on the function of a protein. Amino acids with probabilities of <0.05 are predicted to be affecting protein function (Ng and Henikoff, 2001).

### 2.6 Analysis of T- and B-cell epitopes

A list of experimentally confirmed human epitopes was obtained from the Immune Epitope Database and Analysis Resource (IEDB^4^). The search criteria were human T/B-cell epitopes described either in *Mtb* or *Mtb* H37Rv. A total of 1,470 T-cell epitopes and 472 B-cell epitopes were initially identified. We assigned each epitope to an *H37Rv* gene after inspecting the corresponding bibliographic reference and individual FASTA searches. A total of 237 T-cell epitopes and 44 B-cell epitopes located in non-essential *PE/PPE* genes were excluded from the analysis. In addition, we excluded 70 T-cell epitopes and 7 B-cell epitopes that were unable to identify in the H37Rv genome. Finally, 1,162 T-cell and 421 B-cell epitopes were analyzed. We separately analyzed the characteristics of T-cell epitopes and B-cell epitopes. All genes of H37Rv were divided into antigens (the genes with epitope sequences) and non-antigens (the genes without epitope sequences). The final number of antigens analyzed was 369, including 290 with 1,162 epitope sequences of T-cell and 79 with 421 epitope sequences of B cell. We further divided the sequences of T- and B-cell antigens into epitope sequences (Epi) and non-epitope sequences (NEpi) of the T- and B-cell separately. As many epitopes in the IEDB overlap with other epitopes, 1,162 epitopes of the T-cell were found corresponding to 480 non-overlapping regions, while the 421 epitopes of the B-cell found corresponding to 196 non-overlapping regions in the antigen alignment. These non-overlapping regions in each genome were extracted and analyzed as epitope concatenate sequences, while the left sequences of antigens were analyzed as non-epitope sequences.

The *d_N_/d_S_* were calculated and the ratios were compared between antigens (genes with epitopes) and non-antigens (genes without epitopes) as well as between Epi and NEpi to assess the selective pressure that the host immune system places upon the bacterium to alter antigenic protein regions.

R software (v 4.0.0) was used for statistical analysis (R Core Team, 2020). The Kruskal–Wallis H test was applied to compare the *d_N_/d_S_* between antigens and non-antigens. The Wilcoxon signed-rank test was used to compare the pairwise *d_N_/d_S_* between Epi and NEpi. All statistical tests are paired two-sided, and *p* < 0.05 is considered statistically significant.

## 3 Results

### 3.1 Population structure of *Mtb* strains

**Figure 1:**
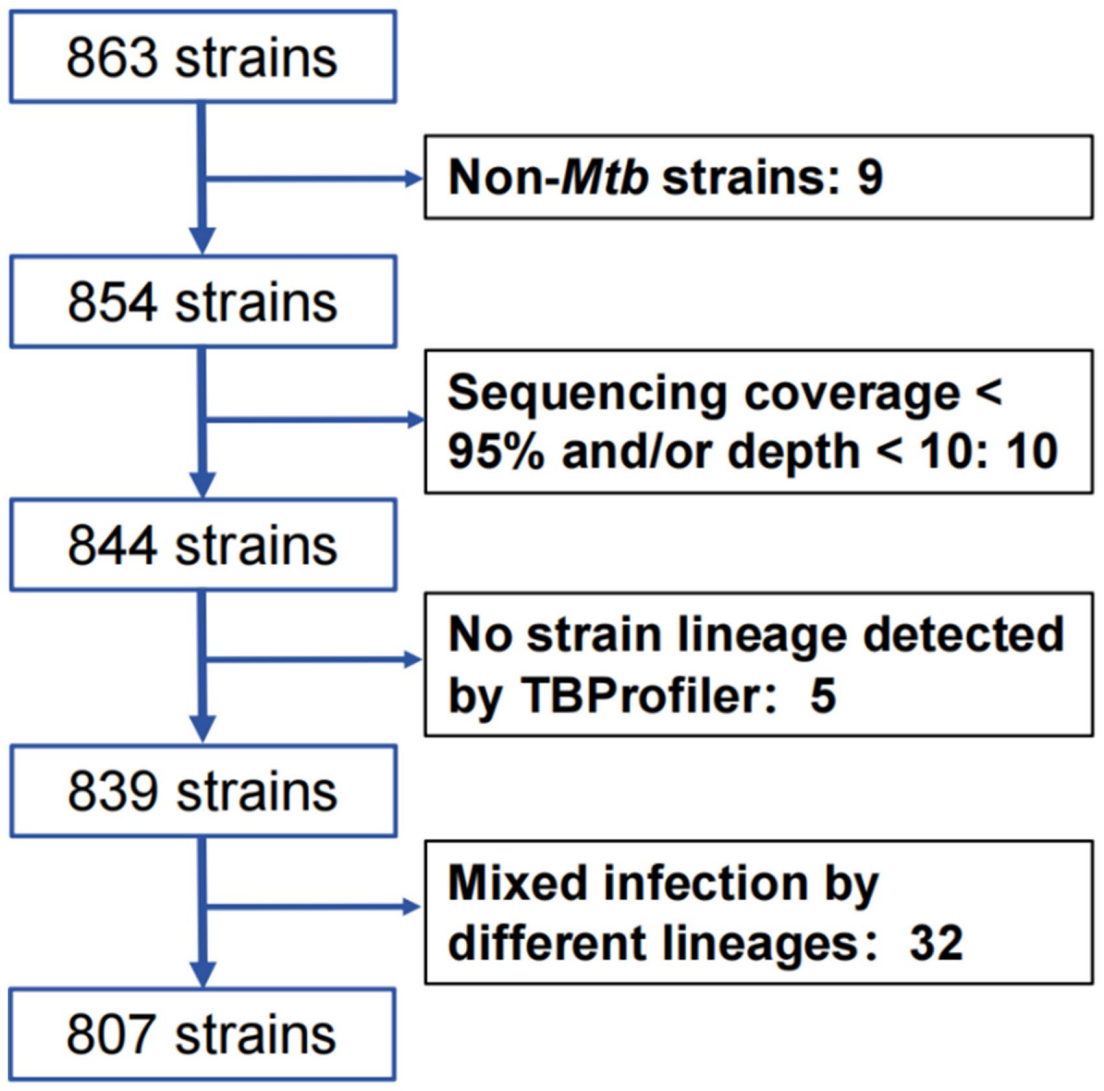
The flow chart of strain collection and filter.

After collecting a total of 863 *Mtb* isolates, WGS was performed, and 807 *Mtb* isolates were included in the final analysis after excluding 32 mixed infection strains, 15 unqualified sequencing strains, and 9 non-*Mtb* strains (Figure 1).

**Figure 2:**
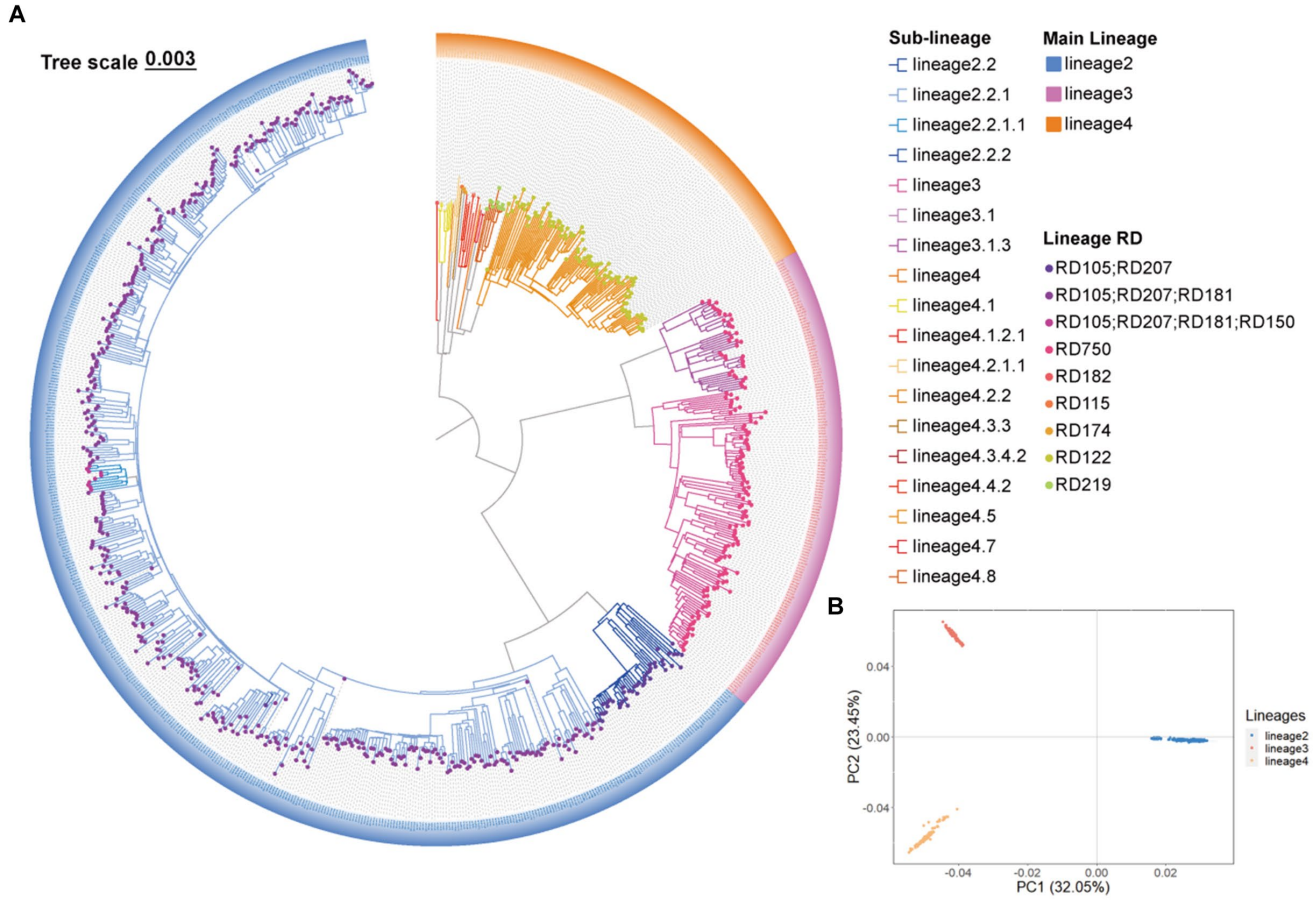
The *Mycobacterium tuberculosis* (*Mtb*) population structure in Southern Xinjiang. **(A)** A maximum likelihood phylogenetic tree of 807 *Mtb* strains in Southern Xinjiang. **(B)** The principal component analysis of 807 *Mtb* strains in Southern Xinjiang.

A total of 44,781 SNPs were identified by mapping 807 genomes to the H37Rv (L4, Euro-American T) reference strain. A phylogenetic tree was constructed using the 44,781 SNPs, and barcode SNP-based genotyping showed that 62.70% (506/807) belong to L2, 19.45% (157/807) belong to L3, and 17.84% (144/807) belong to L4 (Figure 2A). Among the (sub-)lineage strains, L2.2.1, L3, and L4.5 were the most prevalent, which accounted for 55.76% (450/807), 14.13% (114/807), and 13.14% (106/807) of all *Mtb* strains, respectively (Figure 2A; Supplementary Table S2). We further conducted PCA based on the 44,781 SNPs, and the SNPs were classified into three clusters which were similar to that of phylogenetic tree and barcode SNP-based genotyping, further validating the quality of the called SNPs and the reliability of barcode lineages (Figure 2B).

The genotypic resistance of strains was identified by TBProfiler software, including 415 (51.43%) sensitive strains, 147 (18.22%) MDR strains (77 MDR + 2 extensively drug-resistant [XDR] strains +68 pre-extensively drug-resistant [Pre-XDR] strains), 65 (8.05%) HR-TB, 42 (5.20%) RR-TB, and 138 strains identified as “other” (Supplementary Table S2).

### 3.2 Genes and sites under positive selection

**Figure 3:**
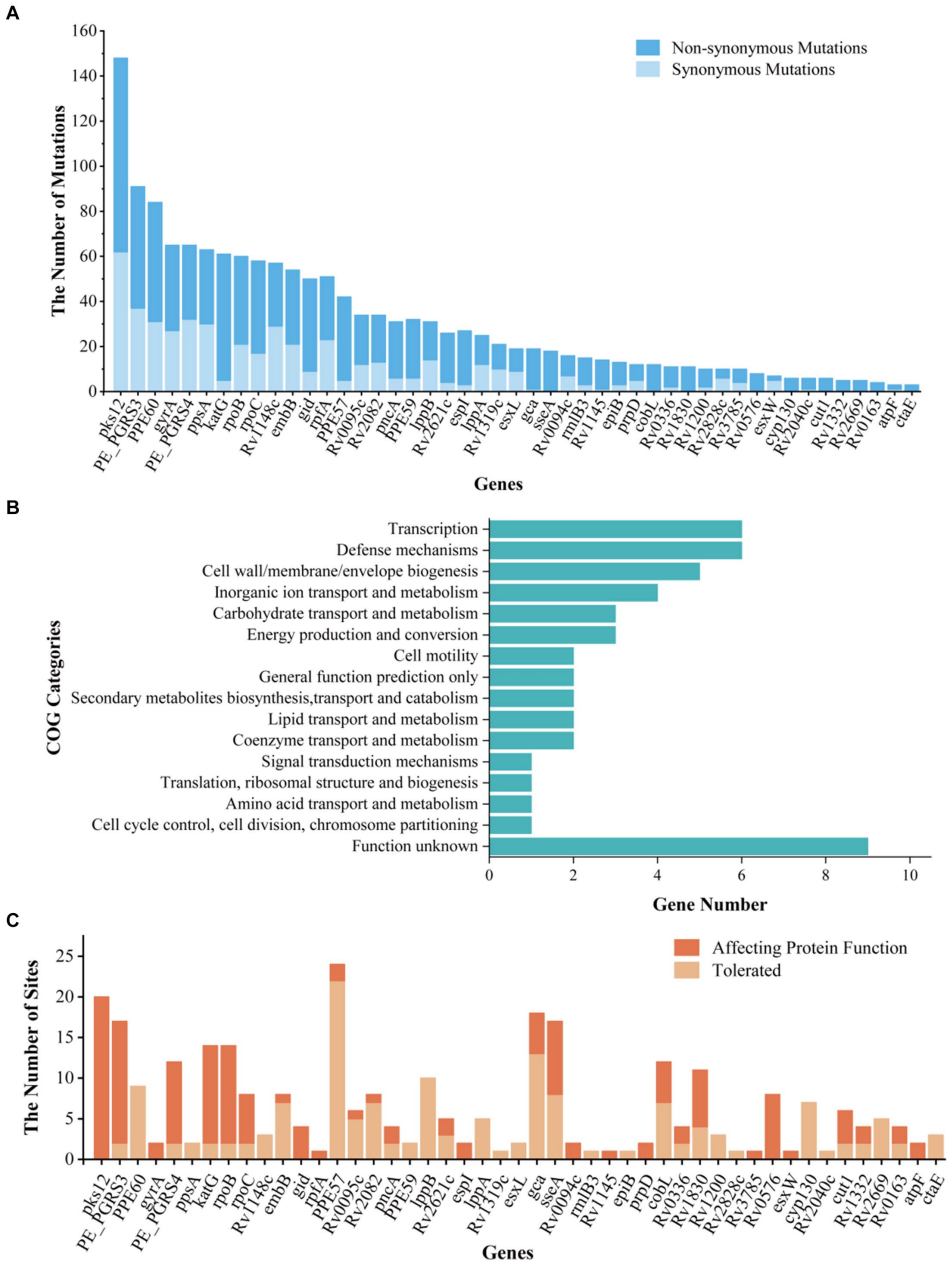
Information, Clusters of Orthologous Groups (COG) categories, and functional prediction of genes and sites under positive selection. **(A)** The list of genes under positive selection. **(B)** COG categories of positively selected genes. **(C)** The prediction of impact on the gene functions of non-synonymous mutations in positively selected codons. “Affecting protein function” represents the number of mutations under positive selection impacting the function of protein; “Tolerate” represents the number of mutations under positive selection affecting the protein function with a small possibility.

The BEB analysis identified 194 codons in 41 genes under positive selection by first comparison (under site models M1a and M2a) and identified 172 codons in 37 genes under positive selection by second comparison (under site models M7 and M8) (Table 1; Figure 3A). A bigger dataset, including 239 positively selected codons in 47 genes, was generated by combining the two comparisons. A total of 1,470 mutations were found in the 47 genes, including 974 non-synonymous mutations, 479 synonymous mutations, and 17 other mutations (e.g., stop codon). Among the 974 non-synonymous mutations, 295 mutations located in 239 codons were under positive selection (Table 1; Supplementary Table S3).

**Table 1:** Information of genes and sites under positive selection in 807 *Mtb* strains.

Gene	Locus	No. SNPs^a^	No. NS^a^	No. S^a^	No. selected codons^a^	Gene product	First comparison	Second comparison
*gyrA*^a^ ^,^^b^	*Rv0006*	65	38	27	1	DNA gyrase subunit A	M1a_M2a	–
*Rv0094c*	*Rv0094c*	16	9	7	2	Hypothetical protein	M1a_M2a	M7_M8
*Rv0095c* ^a^	*Rv0095c*	34	22	12	6	Hypothetical protein	M1a_M2a	M7_M8
*gca*	*Rv0112*	19	18	1	17	GDP-mannose 4,6-dehydratase	M1a_M2a	M7_M8
*Rv0163*	*Rv0163*	4	4	0	3	Hypothetical protein	M1a_M2a	M7_M8
*PE_PGRS3* ^a^	*Rv0278c*	91	54	37	12	PE-PGRS family protein PE_PGRS3	M1a_M2a	M7_M8
*PE_PGRS4* ^a^	*Rv0279c*	65	33	32	9	PE-PGRS family protein PE_PGRS4	M1a_M2a	M7_M8
*Rv0336*	*Rv0336*	11	9	2	3	Hypothetical protein	M1a_M2a	M7_M8
*Rv0576*	*Rv0576*	8	8	0	7	Transcriptional regulator	M1a_M2a	M7_M8
*rpoB* ^a^ ^,^ ^b^	*Rv0667*	60	39	21	7	DNA-directed RNA polymerase subunit beta	M1a_M2a	M7_M8
*rpoC* ^a^ ^,^ ^b^	*Rv0668*	58	41	17	5	DNA-directed RNA polymerase subunit beta	M1a_M2a	M7_M8
*rpfA*	*Rv0867c*	51	28	23	1	Resuscitation-promoting factor RpfA	M1a_M2a	M7_M8
*prpD*	*Rv1130*	12	7	5	2	2-methylcitrate dehydratase	–	M7_M8
*Rv1145*	*Rv1145*	14	13	1	1	Transmembrane transport protein	–	M7_M8
*Rv1148c* ^a^	*Rv1148c*	57	28	29	3	Hypothetical protein	M1a_M2a	–
*esxL*	*Rv1198*	20	10	9	2	ESAT-6 like protein EsxL	M1a_M2a	M7_M8
*Rv1200*	*Rv1200*	10	8	2	2	Integral membrane transport protein	M1a_M2a	M7_M8
*cyp130*	*Rv1256c*	7	6	0	6	Cytochrome P450 Cyp130	M1a_M2a	M7_M8
*atpF*	*Rv1306*	3	2	1	1	ATP synthase subunit B	–	M7_M8
*Rv1319c*	*Rv1319c*	21	11	10	1	Adenylate cyclase	M1a_M2a	–
*Rv1332*	*Rv1332*	5	5	0	3	Transcriptional regulator	M1a_M2a	M7_M8
*cut1*	*Rv1758*	6	6	0	6	Cutinase	M1a_M2a	M7_M8
*Rv1830* ^a^	*Rv1830*	11	11	0	11	HTH-type transcriptional regulator	M1a_M2a	–
*katG* ^a^ ^,^ ^b^	*Rv1908c*	62	56	5	11	Catalase-peroxidase	M1a_M2a	M7_M8
*Rv2040c*	*Rv2040c*	7	5	1	1	Sugar ABC transporter permease	–	M7_M8
*pncA* ^a^ ^,^ ^b^	*Rv2043c*	33	25	6	2	Pyrazinamidase/nicotinamidase PncA	M1a_M2a	M7_M8
*pks12* ^a^	*Rv2048c*	148	86	62	14	Polyketide synthase	M1a_M2a	M7_M8
*cobL*	*Rv2072c*	12	12	0	12	Precorrin-6Y C(5,15)-methyltransferase	M1a_M2a	–
*Rv2082*	*Rv2082*	34	21	13	8	Hypothetical protein	M1a_M2a	–
*ctaE*	*Rv2193*	3	3	0	2	Cytochrome C oxidase subunit III	M1a_M2a	M7_M8
*lppA* ^a^	*Rv2543*	26	13	12	4	Lipoprotein LppA	M1a_M2a	M7_M8
*lppB* ^a^	*Rv2544*	31	17	14	10	Lipoprotein LppB	M1a_M2a	M7_M8
*Rv2621c*	*Rv2621c*	27	22	4	5	Transcriptional regulator	M1a_M2a	M7_M8
*Rv2669*	*Rv2669*	5	5	0	3	GCN5-like N-acetyltransferase	M1a_M2a	M7_M8
*Rv2828c*	*Rv2828c*	10	4	6	1	Hypothetical protein	M1a_M2a	–
*ppsA*	*Rv2931*	63	33	30	2	Phthiocerol synthesis polyketide synthase type I PpsA	M1a_M2a	M7_M8
*sseA* ^a^	*Rv3283*	19	18	0	16	Thiosulfate sulfurtransferase SseA	M1a_M2a	–
*PPE57* ^a^	*Rv3425*	42	37	5	15	PPE family protein PPE57	M1a_M2a	M7_M8
*PPE59* ^a^	*Rv3429*	32	26	6	1	PPE family protein PPE59	M1a_M2a	–
*rmlB3*	*Rv3468c*	15	12	3	1	dTDP-glucose 4,6-dehydratase	M1a_M2a	–
*PPE60* ^a^	*Rv3478*	86	53	31	7	PE family protein PPE60	M1a_M2a	–
*esxW*	*Rv3620c*	8	2	5	1	ESAT-6 like protein EsxW	–	M7_M8
*epiB*	*Rv3784*	14	10	3	1	dTDP-glucose 4,6-dehydratase	–	M7_M8
*Rv3785*	*Rv3785*	10	6	4	1	Hypothetical protein	M1a_M2a	
*embB* ^a^ ^,^ ^b^	*Rv3795*	54	33	21	6	Arabinosyltransferase B	M1a_M2a	M7_M8
*espI*	*Rv3876*	27	24	3	1	ESX-1 secretion-associated protein EspI	M1a_M2a	M7_M8
*gid* ^a^ ^,^ ^b^	*Rv3919c*	54	41	9	3	16S rRNA (guanine(527)-N(7))-methyltransferase RsmG	M1a_M2a	M7_M8

No, number.

a The positively selected genes have identified in other studies.

b Resistance-associated genes.

We then conducted an intensive study on the 47 genes under positive selection from different aspects. First, based on COG categories, the 47 genes under positive selection were divided into 15 categories. The genes associated with transcription (*Rv0576*, *rpoB*, *rpoC*, *Rv2621c*, *Rv2669*, and *Rv1830*), defense mechanisms (*Rv0094c*, *Rv0095c*, *Rv0336*, *cyp130*, *esxW*, and *Rv1148c*), and cell wall/membrane/envelope biogenesis (*gca*, *cut1*, *epiB*, *embB*, and *rmlB3*) were dominant (Figure 3B), suggesting that the selection pressure in these genes lead to their function changes. Second, 7 out of the 47 genes were drug resistance-associated, and 51 mutations occurring in 35 positive selection codons were found to be under positive selection (Table 1; Supplementary Table S3). According to the list of resistance mutations released by World Health Organization (2021), 31 out of the 51 mutations were considered to be drug resistance-associated, including *katG* (D94A, N138H, N138S, S140G, W191R, and S315T; isonizad), *rpoB* (L430P, Q432E, Q432L, D435V, D435Y, D435E, H445D, H445R, H445Q, S450L, and I491F; rifampicin), *rpoC* (G332R and I491T; rifampicin), pncA (G97R and G97D; pyrazinamide), *embB* (M306L, M306I, D328G, G406C, G406D, Q497P, and D1024N; ethambutol), *gid* (E92D; streptomycin), and *gyrA* (D94N, D94G; quinolones). There were still 23 mutations under positive selection in the drug-resistant genes, which were considered to be not associated with resistance.

SIFT was used to predict the functional impact caused by the 295 non-synonymous mutations in 239 positively selected codons of 47 genes (Figure 3C). Among them, 145 mutations were predicted to affect protein function, while the remaining 150 mutations were predicted to be with a small probability of affecting protein function (Figure 3C; Supplementary Table S3).

### 3.3 Special genes and loci under positive selection in MDR strains

**Figure 4:**
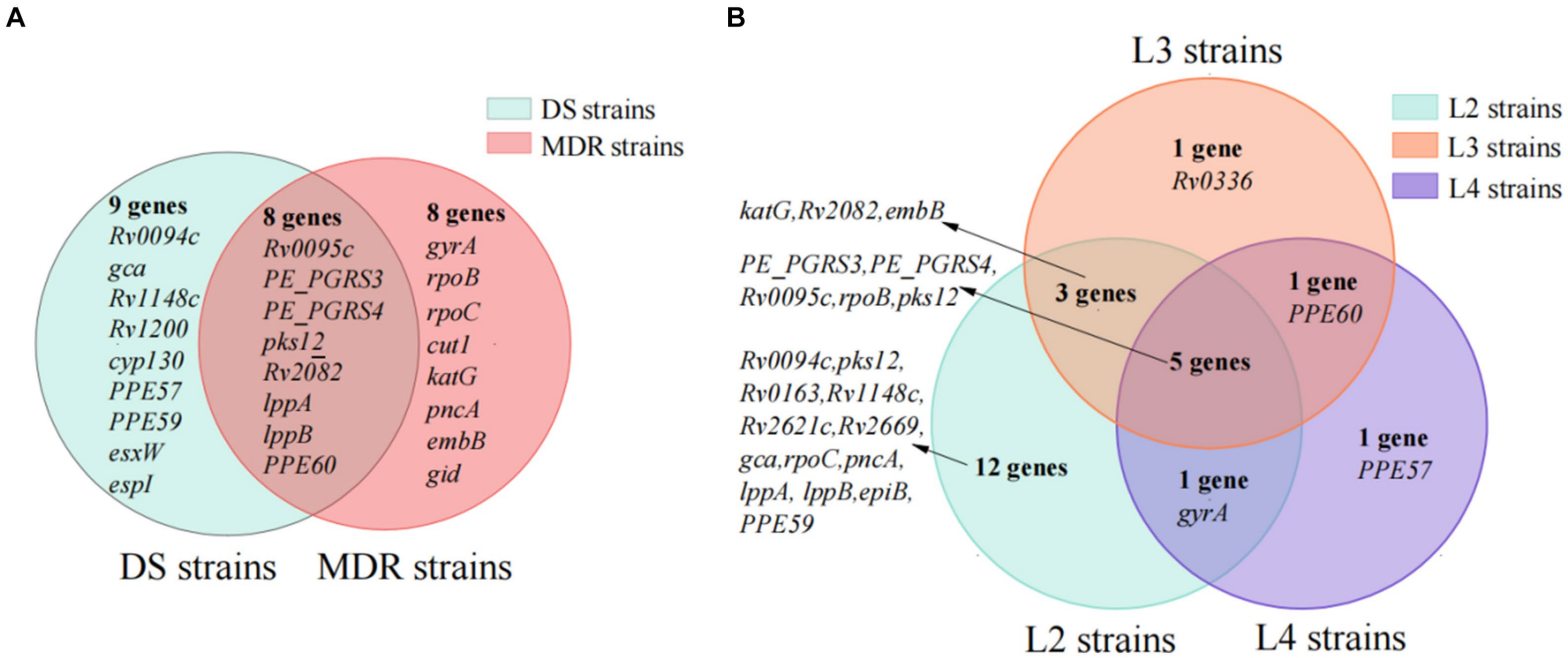
The subset of genes under positive selection in special type of strains. **(A)** The subset of genes in multi-drug-resistant (MDR) and drug-susceptible (DS) strains. **(B)** The subset of genes in lineage2, lineage3, and lineage4 strains.

The genes under positive selection in 147 MDR strains and 415 DS strains were identified by site models (M2a and M8) with PAML. The comparison results between MDR and DS strains showed that, for the codons under positive selection, 35 in 8 genes (*Rv0095c*, *Rv0278c*, *Rv0279c*, *pks12*, *Rv2082*, *lppA*, *lppB*, and *PPE60*) were both in MDR strains and DS strains; 28 in 8 genes (*gyrA*, *rpoB*, *rpoC*, *katG*, *pncA*, *embB*, *gid*, and *cut1*) were in MDR strains but not in DS strains; and 33 in 9 genes were in DS strains but not in MDR strains (Figure 4A; Supplementary Table S4).

The 28 codons under positive selection in 8 genes in MDR but not in DS strains included 23 codons in 7 drug resistance-associated genes (*gyrA*, one codon; *rpoB*, seven codons; *rpoC*, five codons; *katG*, three codons; *pncA*, two codons; *embB*, four codons; and *gid*, one codon) and 5 codons in *cut1* gene. Of the 43 mutations occurring at the 28 codons, 27 were drug resistance-associated, 2 were drug resistance compensatory (*rpoC*_P434R and *rpoC*_V483G), and the remaining 14 mutations (*rpoB*_D435V, *rpoB*_R552S, *rpoC*_P434T, *rpoC*_I491V, *rpoC*_V1039A, *katG*_R463L, *gid*_E92P, *pncA*_A146T, *pncA*_A146E, *cut1*_R6G, *cut1*_F66L, *cut1*_V72A, *cut1*_G119A, and *cut1*_Q164H) have uncertain roles in drug resistance, according to the catalog of WHO (Supplementary Table S4).

The *cut1* gene, which was not reported to be drug resistance-associated, was identified as being under positive selection only in MDR strains, indicating its potential correlation with drug resistance. There were five positively selected codons in the *cut1* gene, of which codon 66 showed the strongest positive selection signals with 9.25 by the site model M8 and 9.03 by site model M2a. Among five codons, *cut1*_F66L, *cut1*_V72A, and *cut1*_Q164H were predicted to affect protein function by SIFT (prediction score < 0.05).

### 3.4 Special genes and loci under positive selection in different lineages

In the present study, 157 out of 807 (19.45%) *Mtb* strains belonged to L3, which was higher than that from other areas in China. We further identified genes under positive selection in L3 strains but not in the L2 and L4 strains to verify the hypothesis that certain positive selections are contributing to the advantage of L3 strains in Southern Xinjiang. Under site models M2a and M8, there were 21, 10, and 8 genes under positive selection in L2, L3, and L4 strains, and 17 codons in 5 genes (*Rv0095c*, *PE_PGRS3*, *PE_PGRS4*, *rpoB*, and *pks12*) were all under positive selection in the L2, L3, and L4 strains (Figure 4B; Supplementary Table S5).

In the L3 strains, 31 codons in 10 genes were under positive selection. Among them, one codon in the *Rv0336* gene was identified only in L3 strains but not in L2 and L4 strains, and the other 30 codons in the 9 genes were identified in L2 and/or L4 strains. One codon with two mutations (P496S and P496H) in the *Rv0336* gene was identified under positive selection among L3 strains, but it was predicted not to affect protein function by SIFT (prediction scores were 0.08 and 0.06, respectively).

### 3.5 Selection pressure on epitopes of T and B cells

Among the 239 positively selected codons in 47 genes, seven genes (*rpoB*, *rpfA*, *esxL*, *cyp130*, *katG*, *esxW,* and *espI*) were with T-cell epitopes and three genes (*rpoB*, *rpoC*, and *katG*) were with B-cell epitopes. However, only two codons with three mutations (Ser315Arg and Ser315Thr of *katG*, Thr2Ala of *esxW*) in the T-cell epitopes were found under positive selection. The three mutations in *katG* and *esxW* were predicted to affect protein function with a high probability (all prediction scores = 0).

**Figure 5:**
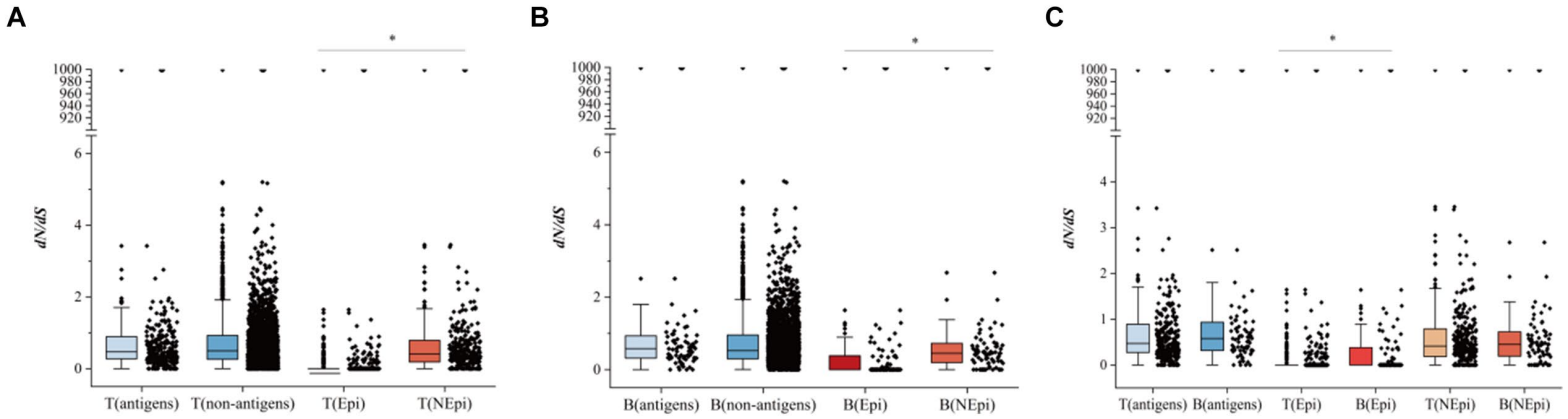
The comparison of *d_N_/d_S_* between different groups. **(A)** The comparison of *d_N_/d_S_* between T-antigens and T-non-antigens, and T-epitopes (T-Epi) and T-non-epitopes (T-NEpi). **(B)** The comparison of *d_N_/d_S_* between B-antigens and B-non-antigens and between B-epitopes (B-Epi) and B-non-epitopes (B-NEpi). **(C)** The comparison of *d_N_/d_S_* between T-cell epitopes and B-cell epitopes. *p < 0.05.

In the present study, we calculated pairwise *d_N_/d_S_* between the Epi and NEpi of T- (or B-) cells, as well as between T- (or B-) cell antigens (genes with epitopes) and non-antigens (genes without epitopes), to assess the selection pressure of T- and B-cell epitopes. There was no significant difference in the *d_N_/d_S_* between the T-antigens and T-non-antigens (Figure 5A) as well as between B-antigens and B-non-antigens (Figure 5B). However, higher *d_N_/d_S_* was found in the T-non-epitopes than in the T-epitopes and also in the B-non-epitopes than in the B-epitopes (Figure 5B). These results suggest that both T- and B-cell epitopes are hyper-conserved.

In addition, comparisons of the *d_N_/d_S_* between the T-antigens and B-antigens and between the NEpi of T-cell and NEpi of B-cell showed no statistical difference, while the *d_N_/d_S_* of the B-cell epitopes was higher than that of the T-cell epitopes (Figure 5C).

## 4 Discussion

This study explored the positively selected genes of *Mtb* strains with large samples from Southern Xinjiang. There were 47 genes with 239 codons under positive selection, and these genes were mostly associated with functions of transcription, defense mechanisms, and cell wall/membrane/envelope biogenesis. MDR and L3 strains showed specific positive evolution features: there were 28 codons (43 mutations) in 8 genes under positive selection in MDR strains but not in DS strains, in which 27 mutations were drug-resistant loci, 9 mutations were non-drug-resistant loci but in drug-resistant genes, 2 mutations were compensatory mutations, and 5 mutations were in unknown drug-resistant gene of *cut1*; there was a codon in *Rv0336* under positive selection in L3 strains but not in L2 and L4 strains. Two codons with three mutations under positive selection (*katG*_S315R, *katG*_S315T, and *esxW*_T2A) were found in the T-cell epitopes. The epitopes of T- and B-cells were both hyper-conserved, especially in the T-cell epitopes. These findings provided clues for understanding the selective evolution of *Mtb*.

Our study showed that L2 was the main lineage and L2.2.1 was the dominant sub-lineage in Southern Xinjiang, which is similar to the findings from other regions of China (Liu et al., 2021; Wu et al., 2021). Among all the lineages, L2 strains were the most widespread globally, and numerous factors influence the spread of L2 strains (Gagneux, 2018). As the oldest strain, the globally extant L2 appears to trace more recent migration events from Southeast Asia and has spread all over the world through human activities and migration (Comas et al., 2013). Following migration, L2 and other lineages may diversify locally and then the dominant strains are gradually fixed in the local area by adaptive evolution (Comas et al., 2013; Chiner-Oms et al., 2022). Some studies indicated that L2 strains were more transmissible and with higher fitness than other lineages, so it is easier to spread and distribute across the world (Reiling et al., 2013; Portevin et al., 2014).

L3 strains were relatively prevalent in China, except for the most prevalent L2 and L4 strains (Liu et al., 2021). In our study, the proportion of L3 strains (19.45%) was higher than the reports of other areas in China (Liu et al., 2021). We found that a codon in *Rv0336* (P496S and P496H) was under positive selection in L3 strains but not in L2 and L4 strains, indicating that these mutations contributed to the survival of L3 strains. *Rv0336* encodes a hypothetical protein, but information on the function of *Rv0336* remains largely unknown (Lew et al., 2011). Further studies are needed to explore their function, especially their contribution to the advantage of L3 strains under positive selection in Southern Xinjiang by integrating the bacteria’s own properties, environment, and host factors (Gagneux, 2018).

Previous literature reported that positive selection pressure drives the evolution of *Mtb* strains (Gagneux, 2018; Liu et al., 2021). Non-synonymous mutations could drive the phenotypic differences. The positive selection genes were evolutionary signatures, which play crucial roles in the fixation of mutations in bacterial populations. Several studies have identified the positively selected genes with important functions, illustrating the ongoing positive selection in the *Mtb* population (Farhat et al., 2013; Zhang et al., 2013; Liu et al., 2021). Our study identified 239 positively selected codons in 47 genes, and these genes focus on the functions of transcription, defense mechanisms, and cell wall/membrane/envelope biogenesis. SIFT revealed that most of these mutations were with a high probability of affecting protein function. Genes under positive selection enriched for transcriptional function have been reported (Liu et al., 2022), and the responses of *Mtb* at the level of transcription and translational regulation will adapt themselves to the external and host environmental conditions (Ghosh et al., 2016). Functional enrichment analysis indicated that positively selected genes are highly enriched for transcriptional regulators, especially *resR* (Liu et al., 2022). Further experiments found that *resR* mutants were approximately 20% longer and approximately 5% wider than the wild-type cells and had a thickened cell envelope, suggesting that mutations of *resR* have functional consequences on bacterial size control under standard growth conditions (Liu et al., 2022). In addition, genes of defense mechanisms and cell wall/membrane/envelope biogenesis are important for *Mtb*, influencing its phenotypes, such as virulence and drug resistance (Ryndak et al., 2008; Majumdar et al., 2012). Some studies in *Mtb* have often excluded *PE/PPE/PE-PGRS* genes from analysis because of their high genetic similarity. It is reported that *PE/PPE/PE-PGRS* proteins in *Mtb* exhibit distinct features, including their abundance, cell wall localization, sequence variation, immunogenicity, and potential roles in virulence (Phelan et al., 2016; Gómez-González et al., 2023). Based on the findings of other studies, we analyzed the *d_N_/d_S_* of several essential *PE/PPE* genes (*PE-PGRS3*, *PE-PGRS4*, *PE-PGRS17*, *PPE57*, *PPE59*, and *PPE60*) and excluded non-essential *PE/PPE/PE-PGRS* family genes, to explore the evolutionary characteristics (Phelan et al., 2016). We found that *PE_PGRS3*, *PE_PGRS4*, *PPE57*, and *PPE59* were under positive selection in 807 *Mtb* strains, which was consistent with other studies (Phelan et al., 2016). There was evidence that some *PE/PPE/PE-PGRS* genes were undergoing purifying selection pressure, which provide potential insights into the use of *PE/PPE/PE-PGRS* genes. For example, the *PPE57* protein has been found to be a potential antigen for the rational design of an efficient vaccine against *Mtb* (Xu et al., 2015).

This study revealed that seven drug-resistant genes (*katG*, *rpoB*, *rpoC*, *pncA*, *embB*, *gid*, and *gyrA*) were under positive selection, which also reported in other studies (Farhat et al., 2013; Zhang et al., 2013; Liu et al., 2021, 2022). Among the seven drug-resistant genes, 35 codons with 51 mutations were under positive selection, while 31 out of 54 mutations were drug resistance-associated loci (World Health Organization, 2021). A comparison of the difference of positively selected genes between MDR and DS strains was further performed, and seven drug resistance-associated genes were identified to be under positive selection only in MDR but not in DS strains. Previous studies showed that antibiotics contribute to the positive selection of antibiotic resistance-associated genes, manifesting as a change in biological functions (Osório et al., 2013; Chiner-Oms et al., 2022; Liu et al., 2022).

It was noteworthy that the *cut1* gene with five codons under positive selection was only in the MDR strains but not in DS strains. *cut1* (*Rv1758*) encodes cutinase, which shows potential catalytic activity and is associated with the function of cell wall/membrane/envelope biogenesis (Monu and Meena, 2016). The association between *cut1* and drug resistance has not been reported. Moreover, the impact of mutations in the *cut1* gene on the function and phenotype of *Mtb* is still unclear and deserves further research. Two codons in *rpoC* under positive selection were compensatory mutations (*rpoC*_P434R and *rpoC*_V483G) for rifampicin pressure, indicating that compensatory evolution improves the adaptability of *Mtb* (Wang et al., 2020). According to the catalog of WHO, for the 14 positively selected mutations in drug resistance-associated genes that have uncertain roles in drug resistance, mutations of *katG*_R463L and *gid*_E92P were reported to be associated with the Beijing genotype and not with isoniazid and streptomycin resistance, respectively (Wan et al., 2020); *rpoB* D435V and *rpoC*_I491V were found in rifampicin-resistant isolates (Vargas et al., 2020; Wan et al., 2020; Wang et al., 2020); *pncA*_A146T was found in pyrazinamide-resistant isolates (Che et al., 2015; Vargas et al., 2020; Wang et al., 2020; World Health Organization, 2021); *rpoC*_P434T, *rpoC*_V1039A, and *pncA*_A146E and the five mutations in the *cut1* were newly identified.

This study found that the T-cell epitopes were hyper-conserved in *Mtb* strains, which is similar to previous studies (Comas et al., 2010). The possible explanation for the hyper-conservation of T-cell epitopes is that *Mtb* adopts a strategy of immune escape, and the regions encoding proteins are unlikely to mutate without fitness loss compensation and are essential for virulence (Comas et al., 2010; Coscolla et al., 2015; Gagneux, 2018). Although the human T-cell epitopes in *Mtb* demonstrated conservative roles, there were two codons with three mutations (Ser315Arg and Ser315Thr of *katG*, and Thr2Ala of *esxW*) in the T-cell epitopes under positive selection, exhibiting higher genetic diversity in specific T-cell epitopes. It was reported that *esxW* Thr2Ala is associated with increased transmissibility in Lineage 2 (2.2.1) *Mtb* strains (Holt et al., 2018; Brown et al., 2021), and *katG*_S315R and *katG*_S315T are both highly reliable markers for isoniazid resistance (World Health Organization, 2021). We speculate that *Mtb* strains bearing *katG*315 mutations are beneficial for the pathogen escaping from isoniazid pressure and human T-cell recognition (Coscolla et al., 2015), and the addition of *esxW* Thr2Ala increases the speed of the spread of isoniazid-resistant *Mtb*. Moreover, the epitope region that *katG*_315 may be used as a potential target for developing new tools to prevent the transmission of isoniazid-resistant TB. Similar to the T-cell epitopes, B-cell epitopes were more hyper-conserved than the non-epitope regions. However, the genetic diversity of B-cell epitopes was higher than T-cell epitopes. One of the explanations may be due to the small number of B-cell epitopes compared to that of T-cell epitopes (421 vs. 1,162) while both types of epitopes under similar genetic diversity, and detail mechanisms for the difference need further exploration.

Generally, the samples were collected from three areas in Southern Xinjiang, which may be a limitation in inferring the findings to the entire Xinjiang region. The lack of annotated epitopes for a given gene in IEDB does not necessarily imply that it is non-antigenic, which may be a limitation in the comparison between genes with epitopes and those without epitopes. Due to the high repetition of *PE/PPE/PE-PGRS* genes in the *Mtb* genome, the detection of positive selection within these genes can be influenced by non-specific mapping.

## 5 Conclusion

Overall, this study revealed the ongoing selective evolution of *Mtb* in Southern Xinjiang of China and identified 47 genes with 239 codons under positive selection in 807 *Mtb*. We found that MDR and L3 strains showed specific positive evolution features: Nine mutations that were reported not to be associated with resistance in drug-resistant genes were under positive selection in MDR but not in DS strains; five mutations in the *cut1* gene which were not reported to be associated with drug resistance were under positive selection in MDR but not in DS strains; and there was a codon in *Rv0336* under positive selection in L3 strains but not in L2 and L4 strains. The findings provide important information for future research on these significant mutations related to TB control.

## Data availability statement

The original contributions presented in the study are included in the article/Supplementary material, further inquiries can be directed to the corresponding authors.

## Ethics statement

The studies involving humans were approved by Human Ethics Committee of the Eighth Affiliated Hospital of Xinjiang Medical University. The studies were conducted in accordance with the local legislation and institutional requirements. The participants provided their written informed consent to participate in this study. Written informed consent was obtained from the individual(s) for the publication of any potentially identifiable images or data included in this article.

## Author contributions

LD: Data curation, Formal analysis, Methodology, Software, Visualization, Writing – original draft. QW: Data curation, Writing – review & editing. HL: Software, Writing – review & editing. YJ: Writing – review & editing. MX: Data curation, Investigation, Writing – review & editing. YX: Software, Writing – review & editing. TY: Data curation, Investigation, Writing – review & editing. SY: Software, Writing – review & editing. DY: Data curation, Investigation, Writing – review & editing. ML: Writing – review & editing. LZ: Writing – review & editing. XZ: Writing – review & editing. KW: Funding acquisition, Project administration, Writing – review & editing. GH: Formal analysis, Methodology, Writing – review & editing. XM: Data curation, Writing – review & editing. GL: Formal analysis, Methodology, Software, Writing – review & editing.

## Footnotes

^1^ https://www.chiplot.online/

^2^ https://www.ncbi.nlm.nih.gov/COG

^3^ https://sift.bii.a-star.edu.sg/

^4^ http://www.iedb.org/
